# Extensive nuclear reprogramming and endoreduplication in mature leaf during floral induction

**DOI:** 10.1186/s12870-019-1738-6

**Published:** 2019-04-11

**Authors:** Stefania Del Prete, Anne Molitor, Delphine Charif, Nadia Bessoltane, Ludivine Soubigou-Taconnat, Cécile Guichard, Véronique Brunaud, Fabienne Granier, Paul Fransz, Valérie Gaudin

**Affiliations:** 1 Institut Jean-Pierre Bourgin, INRA, AgroParisTech, CNRS, Université Paris-Saclay, INRA Centre de Versailles-Grignon, Bât. 2, RD10 Route de Saint-Cyr, 78000 Versailles, France; 2 Institute of Plant Sciences Paris-Saclay (IPS2), CNRS, INRA, Université Paris-Sud, Université Evry, Université Paris-Saclay, Bâtiment 630, Plateau du Moulon, 91192 Gif-sur-Yvette, France; 3 Institute of Plant Sciences Paris-Saclay (IPS2), CNRS, INRA, Université Paris Diderot, Sorbonne Paris-Cité, Bâtiment 630, Plateau du Moulon, 91192 Gif-sur-Yvette, 91405 Orsay, France; 4 Swammerdam Institute for Life Sciences, University of Amsterdam, 1098XH Amsterdam, The Netherlands

**Keywords:** Floral transition, Leaf, Arabidopsis, Transcription, Non-coding RNA, Transcription factors, DNA motif, Endoreduplication

## Abstract

**Background:**

The floral transition is a complex developmental event, fine-tuned by various environmental and endogenous cues to ensure the success of offspring production. Leaves are key organs in sensing floral inductive signals, such as a change in light regime, and in the production of the mobile florigen. *CONSTANS* and *FLOWERING LOCUS T* are major players in leaves in response to photoperiod. Morphological and molecular events during the floral transition have been intensively studied in the shoot apical meristem. To better understand the concomitant processes in leaves, which are less described, we investigated the nuclear changes in fully developed leaves during the time course of the floral transition.

**Results:**

We highlighted new putative regulatory candidates of flowering in leaves. We observed differential expression profiles of genes related to cellular, hormonal and metabolic actions, but also of genes encoding long non-coding RNAs and new natural antisense transcripts**.** In addition, we detected a significant increase in ploidy level during the floral transition, indicating endoreduplication.

**Conclusions:**

Our data indicate that differentiated mature leaves, possess physiological plasticity and undergo extensive nuclear reprogramming during the floral transition**.** The dynamic events point at functionally related networks of transcription factors and novel regulatory motifs, but also complex hormonal and metabolic changes.

**Electronic supplementary material:**

The online version of this article (10.1186/s12870-019-1738-6) contains supplementary material, which is available to authorized users.

## Background

The transition to flowering is a decisive developmental event in the plant life cycle for reproductive success. The general understanding highlights a fine-tuned process involving a complex interplay between environmental and endogenous cues. Signals are perceived and decoded according to the plants’ lifestyle, and lead to a cascade of dramatic morphological changes at the meristem level, to produce floral organs [1, 2].

Photoperiod is a major parameter controlling the transition to flowering with intricate phototropic effects and links with the circadian clock. The light signal, perceived in the leaves, triggers the accumulation of metabolites and regulators, such as the well-conserved FLOWERING LOCUS T (FT) protein, whose expression is under the control of the CONSTANS (CO), a zinc finger transcription factor involved in photoperiod pathway [3]. Their export, as systemic florigen signals via the vasculature to the distant shoot apical meristem (SAM), activates floral homeotic genes [4–6]. Described as a quantitative long-day (LD) species, the photoperiodic property of Arabidopsis species was exploited to induce synchronous flowering by exposure to a single LD or a single displaced short-day (SD), providing a convenient experimental inductive system [7]. Besides photoperiod, other regulatory pathways partake to the vegetative-to-reproductive switch control [8–10].

From transcriptional and chromatin-based mechanisms, to alternative splicing and post-translational regulation, numerous regulatory levels participate to the control of the floral transition [9, 11–15] and its main actors, which have been gathered in the Flowering-Interactive Database (FLOR-ID) [16]. Besides protein regulators involved in developmental transitions, an increasing number of studies have highlighted the regulatory functions of long non-coding RNAs (lncRNAs) [17, 18]. In response to vernalization, the lncRNAs *COLDAIR*, *COLDWRAP*, *COOLAIR*, and *Antisense Long* participate to the fine regulation of the key MADS-box floral repressor *FLOWERING LOCUS C (FLC)* via modifications of *FLC* chromatin environment [19–23]. Recently, *FLORE*, a Natural Antisense Transcript (NAT) of *CYCLING DOF FACTOR5* (*CDF5*) was shown to positively regulate flowering time, repressing *CDF* TFs (*CDF1*, *CDF3*, *CDF5*), and subsequently increasing *FT* expression [24]. LncRNAs are versatile regulators involved in transcriptional gene regulation*,* in guiding or scaffolding protein complexes involved in chromatin organization and gene regulation, or even in post-transcriptional regulatory mechanisms [25]. Due to their large number estimated at several thousands and their diversity (intergenic ncRNAs, intronic ncRNAs, antisense RNAs, *cis* or *trans* NATs…) [19, 23, 26, 27], their functional annotations and roles in developmental phase transitions remain poorly explored.

The transition to flowering is an integrated process at the scale of the whole plant. Few studies analyzed the transcriptional behaviors of meristematic and root tissues during the floral transition at the genome level [28–32]. Early studies identified few CO targets differentially expressed during flowering in leaves, among which *FT* [33] which was identified as the major CO target involved in the SD to LD shift response [34]. *FT* is referred as a flowering integrator with *TWIN SISTER OF FT* (*TSF*) [35, 36]. Subsequent studies increased the number of associated genes involved in the leaf response during flowering (see FLOR-ID overview and references therein). However, the dynamics of genome-wide transcriptomes in leaves during the floral transition has not been reported despite the key functions of leaves as receptors of the inductive photoperiodic signal and producer of florigenic molecules. Here, by exploiting the inductive response to a long-day (LD) shift [7] and disconnecting leaf growth or developmental responses from the floral inductive response, we performed a large transcriptome analysis, and identified novel loci and regulatory elements involved in flowering in mature leaves. The transcriptome dataset enabled us to highlight molecular events, providing new insights into transcriptional reprogramming in leaves accompanying the floral transition. Observations of endoreduplication events supported transcriptome data and suggested a novel function in flowering.

## Results

### Flowering and organ growth in mature leaves

**Fig. 1:**
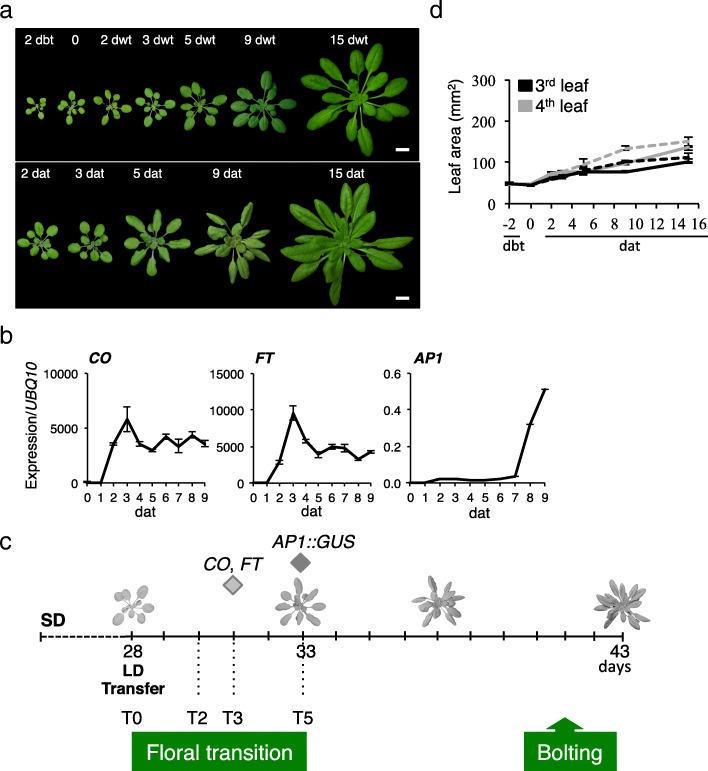
Floral transition induced by a SD-LD switch in *A. thaliana*. **a** Col-0 plants were grown in SD for 4 weeks, kept in SD (upper row) or transferred in LD (bottom row) conditions. Day before transfer (dbt), day without transfer (dwt), day after transfer (dat). Scale bars, 1 cm. **b** Expression of key flowering time genes. Experimental values are mean ± SEM. **c** Schema with developmental events. The *CO* and *FT* expression peaks are indicated, whereas the *AP1* expression initiation was considered as the upper limit of the floral transition (diamonds). T0, day of transfer in LD. **d** Area measurement of the third and fourth rosette leaves. SD conditions (continuous line), transfer in LD (dash line). Experimental values are mean ± SEM

Our experimental system was based on a photoperiodic shift from SD to LD, which induces a synchronized flowering appropriate to analyze the floral transition (Fig. 1a, Additional file 1: Figure S1a). The floral transition window was determined by the onset of the expression of *CO* and *FT,* two early markers for flowering and of *APETALA 1* (*AP1),* an early marker of the floral meristem identity (Fig. 1b, c). *CO* and *FT* expression rapidly increased after the transfer in LD, and reached a maximum at 3 days after transfer (dat), with a slight delay for *FT* consistently with the primary activating role of *CO* (Fig. 1b). Although *AP1 mRNA* transcripts were detected at 7 dat, *AP1* expression was slightly earlier (at 5 dat) in meristematic cells when using an *AP1::GUS* transgenic line, suggesting a completion of the floral transition at that time (Fig. 1b, c, Additional file 1: Figure S1b).

To complete our characterization, we monitored organ growth accompanying the photoperiodic shift. No significant difference was detected in the rosette size during the window of 0–5 dat between the continuous SD and SD-LD conditions, whereas the first six rosette leaves presented different behaviors (Fig. 1d, Additional file 2: Figure S2). The size of the first two leaves was already established 2 days before transfer (dbt), independently of the photoperiodic conditions. Leaves 3–4 showed no significant differences in growth rate between SD and SD-LD conditions from 0 to 5 dat. Leaves 5–6 presented a higher and continuous growth rate over the 15 days. Thus, during the 5-day floral transition window, developmental and growth processes are arrested in leaves 3–4, which makes this pair of mature leaves appropriate material for investigating the early molecular events associated with floral transition, independent of other developmental or signaling events.

### Ploidy level changes during the floral transition window

**Fig. 2:**
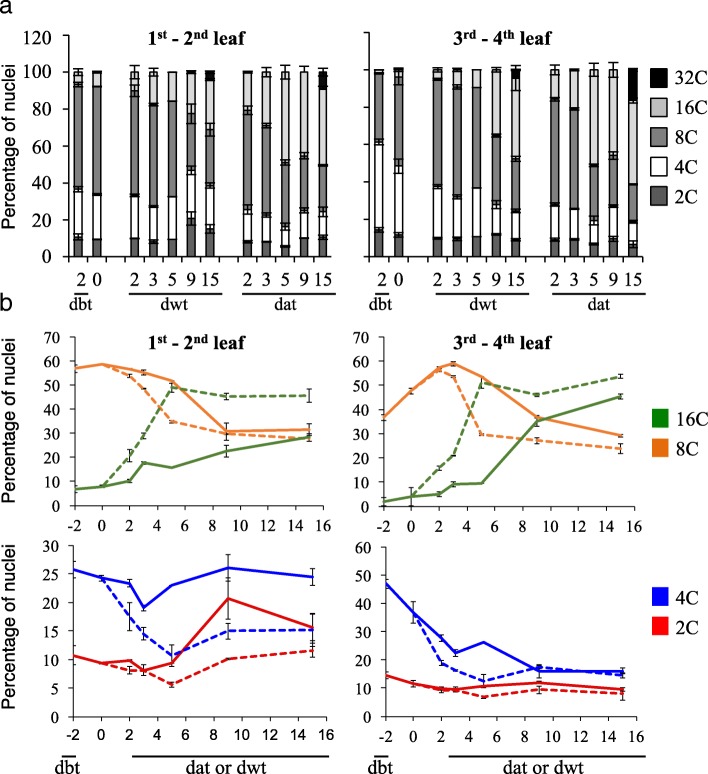
Ploidy dynamics in leaves during the floral transition. Plants were grown 4 weeks in SD, then either, kept in SD or transferred in LD for 15 days. **a** Cumulative distributions. 32C nuclei were only detected at 15 dat. **b** Dynamics of the distribution of the 2C to 16C nuclei. Experimental values are mean ± SEM. dbt, day before transfer. Dwt, day without transfer (continuous line). dat, day after transfer (dash line)

Since plant development is accompanied by endoreduplication, we analyzed the ploidy distribution in leaves 1–4. Independently of the leaf position, age and growth conditions, the percentage of 2C ploidy nuclei was relatively constant (Fig. 2), suggesting a low cell division rate in agreement with the growth analysis. The ploidy levels evolved over time from 4C to 32C. The dynamics was dependent on the leaf position and the growth conditions. The ploidy levels in leaves 3–4 in both conditions suggested that endoreduplication events occurred (8C and 16C nuclei) in response to the LD shift. A population of 32C nuclei was detected at 15 dat and was significantly larger in SD-LD compared to SD conditions. The endoreduplication index evolved from 1.95 ± 0.01 (15 dwt) to 2.35 ± 0.03 (15 dat) in leaves 3–4, whereas it was rather constant in leaves 1–2 (2.08 ± 0.02 at 15 dwt versus 2.04 ± 0.09 at 15 dat). Thus, the photoperiodic SD-LD switch showed a significant increase in endoreduplication in mature leaves, which occurred during the 3–5 dat window of the floral transition, earlier compared to SD (15 dat).

### Major changes in transcription profiles during the inductive shift

To characterize the molecular events during the floral transition, we examined RNA profiles in leaves 3–4 at different time points (T0, T2, T3, and T5) (Fig. 1c, Additional file 3: Figure S3). We identified 20,284 genes expressed at least in one of the four time points, with more than 6000 differentially expressed genes (DEGs) at the largest transition (T0/T2) and specific gene sets at the main T0/T2, T2/T3 and T3/T5 transitions. By assembling a non-redundant dataset of 14,621 long non-coding transcription units (lncTUs) based on TAIR annotations and published datasets, we also identified 531 differentially expressed lncTUs (DE-lncTUs) (Additional file 4: Figure S4, Additional file 5). These data endorse the highly dynamic transcriptional activity in mature leaves in response to the SD-LD switch.

**Fig. 3:**
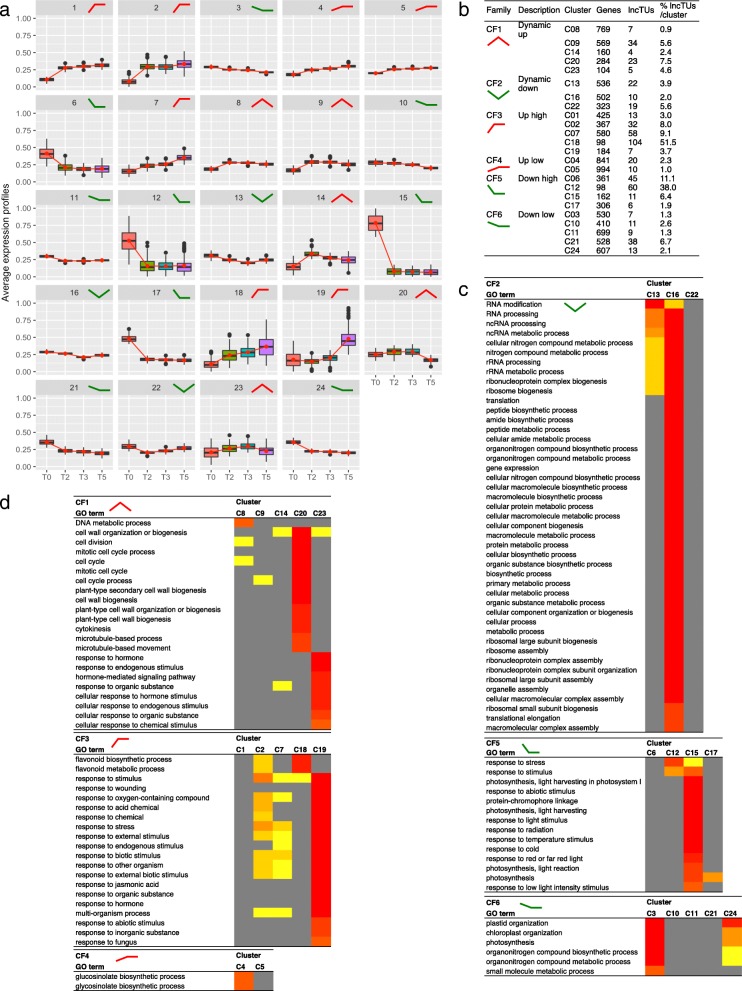
Hierarchical clustering of DEGs and DE-lncTUs using their expression levels. **a** Expression profiles of the 24 clusters. **b** Composition of the clusters and their organization into 6 cluster families (CFs) according to their general expression tendencies. **c**-**d** Heat maps with the biological process GO terms of the clusters per family using SEACOMPARE. Only GO terms with FDR < 10–8 at least in one of the clusters are presented

To characterize the transcriptome profiles, we performed a clustering analysis using different clustering methods and transformation functions (R package coseq). Twenty-four clusters formed by genes and lncTUs (Additional file 6) were identified and further grouped into 6 cluster families (CF1 to CF6) according to expression tendencies (Fig. 3a, b): transiently-up (CF1) or down (CF2), stable high up (CF3) or down (CF5), stable low up (CF4) or down (CF6).

The 24 clusters had specific GO term enrichments, even among cluster families, such as seven clusters (C20, C23, C16, C18, C19, C15, C4, C3) with strong signatures, thus supporting the cluster analysis (Fig. 3c, d, Additional file 7). This classification revealed different processes in mature leaves during the SD-LD response. For instance, C3, C15, C17 and C24 were enriched in down-regulated genes involved in photosynthesis chlorophyll biosynthesis process, light harvesting, or plastid organization, pointing at a reprogramming of the photosynthetic apparatus. C12 and C15 from CF5 and most clusters in CF3 were enriched in stress-associated terms, such as defence response, response to stimuli, response-to-wounding, highlighting a stress association with the changes in photoperiod, light and perturbation of the circadian clock. In accordance, we noticed in C19, C2 and C14, “child” GO terms associated with jasmonic acid, salicylic acid or brassinosteroids, respectively. Secondary metabolites, such as flavonoids (C18) and the defence-related glucosinolates (C4), and carbon metabolism (GO terms such as “glucan catabolic process”, “cellular polysaccharide catabolic process” in C17) were also modified. We observed enrichments in GO terms associated with cell wall, such as “cell wall organization” and “xyloglucan metabolic process” in C14 or “plant-type secondary cell wall biogenesis” and “cellulose metabolic process” in C20. For instance, *XYLOGLUCAN ENDOTRANSGLUCOSYLASE/HYDROLASE 9 (XTH9*) involved in cell wall loosening was up-regulated in C14 (log2 ratio 1.85, FDR 1.28E-3 at T0/T2). These data suggest that the SD-LD switch is accompanied by cell wall remodeling and some cell wall plasticity in the mature leaves in response to environmental changes. Cell wall modifications were reported in roots in response to inorganic phosphate starvation or in hypocotyl in response to light signaling [37]. Such examples of cell wall remodeling in relation to environment signaling remain rarely reported, especially in leaf. We also noticed in C14 a GO term “response to cyclopentenones”, which are fatty acid derivatives with signaling activities. C16 cluster, belonging to CF2 with a transient down-regulation profile, had the highest enrichment terms related to translation, RNA processing and metabolism, suggesting that a strong modification of the protein metabolism at the cellular level is possibly escorting the transition of the metabolic regime occurring in the whole plant during the switch to reproductive phase. Such transient modifications of translation and associated processes were also observed during a cell dedifferentiation and re-differentiation process in Arabidopsis protoplasts [38]. Finally, C20 was enriched in GO terms associated with cell cycle processes. The modifications of the expression of *LGO/SMR1*, *KRP2* and *KRP6* cell cycle inhibitor genes involved in endoreduplication [39], the *CYCA2;3*, a suppressor of endocycles, the major cell-cycle markers, *CDKB2.1*, *CYCA1;1*, and *WEE1,* a negative regulator of the entrance in the M phase [40, 41] were consistent with the onset of the observed endocycles (Additional file 8: Figure S5). In summary, leaf transcriptome during the floral transition revealed major changes in numerous processes, such as carbon and secondary metabolism, signaling events, and endoreduplication.

### Flowering and hormone-related genes are differentially expressed in mature leaves during the floral induction

**Fig. 4:**
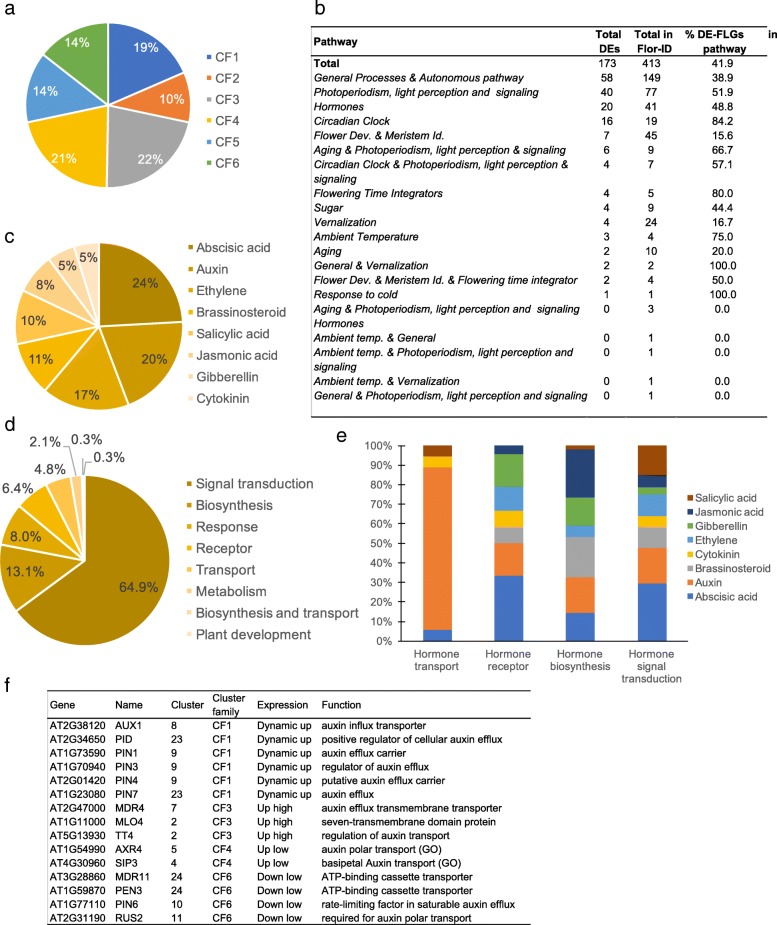
Analyses of genes differentially expressed during the SD-LD switch involved in flowering (FLGs) and hormone responses. **a** Distribution of the DE-FLGs in the different cluster families. **b** Repartition of the DEGs in the regulatory pathways involved in flowering. **c, d** Distribution of DEGs belonging to the AHD according to hormones (**c**) and to the pathways (**d**). **e** Distributions in the 5 main functional pathways. **f** DEGs related to auxin transport

The FLOR-ID core database records genes (FLGs) related to the different regulatory pathways of flowering time and to flower development [16]. To better characterize the molecular events, we analyzed the expression of these genes in mature leaves during the floral transition. We identified a set of 173 DE-FLGs out of the 413 FLGs. These genes were rather evenly distributed among different clusters and regulatory pathways (Fig. 4a, b, Additional file 9). As expected, the major floral integrators were upregulated, such as *FT, TWIN SISTER OF FT* (*TSF*), *BROTHER OF FT* (*BFT*), *CO* and *COL1*. The 173-gene set also included flower meristem identity and flower development (FMI-FD) genes, such as *AP2*, *PETAL LOSS* and *SEPALLATA 4*. Consistently with the experimental design based on a photoperiod shift, most of the FLOR-ID genes classified as associated with the circadian clock were differentially expressed (84.2%). This large number of DEGs from FLOR-ID in mature leaves highlighted how complex and broad the regulatory gene network of the floral transition is.

In the Arabidopsis Hormone Database (AHD) [42], we identified 331 DEGs involved in hormonal regulation from biosynthesis, metabolism, perception, and transport to hormonal responses. C23 was the most enriched cluster with these genes (Additional file 10; Additional file 11: Figure S6). We noticed that genes related to abscisic acid (ABA) and auxin were the most represented ones among the DEGs (Fig. 4c). Genes involved in the ABA biosynthesis were down-regulated in agreement with a repressive role of ABA in flowering [43]. However, the switch also largely impacted genes related to hormone signal transduction such as auxin transport (Fig. 4d, e). Among the DEGs involved in auxin-hormone transport, most genes were up-regulated at least transiently during the switch, such as several PIN members (Fig. 4f), questioning the role of auxin transporters in the flowering time control. Genes involved in gibberellin (GA) biosynthesis were mainly down-regulated, while GAMT2, a methyltransferase involved in the GA metabolism was activated, as well as negative regulators of GA responses (*RGA-LIKE1–2*) (Additional file 11: Figure S6b). Indeed, genes involved in cytokinin (CK) biosynthesis and the *SOB FIVE-LIKE 1, 2* genes (*SOFL1*, *2*), which participate to CK level regulation [44] were activated, whereas genes involved in CK catabolism (*CKK4*, *CKK6*) were down-regulated. Consistently, the type-B ARABIDOPSIS RESPONSE REGULATOR 10 (ARR10) TF, a key player in the CK signaling pathway for the light response and shoot initiation [45], was up-regulated between T0/T5. Beside DEGs involved in one hormonal pathway, 36 DEGs are involved in hormonal crosstalk, with ABA being involved in most of these crosstalk (Additional file 12). Whereas GA is proposed to promote the floral transition and have antagonistic effects with ABA, our data suggest a complex hormonal interplay during the SD-LD switch, with GA, ABA but also new players such as the brassinosteroids and derivative forms, as well as IAA and CKs.

### Novel regulatory actors involved in the SD-LD switch

**Fig. 5:**
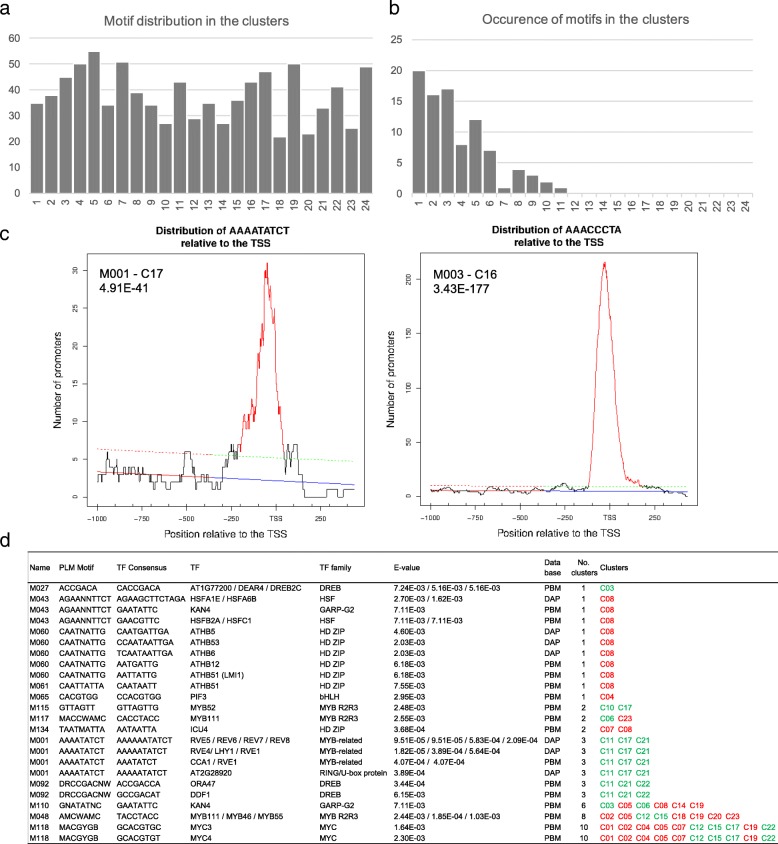
Identification of preferentially located DNA motifs (PLM). **a** Distribution in the 24 clusters of the PLM motifs (≥7 bp). **b** Occurrence of the motifs (≥7 bp) in the clusters. For instance, there are 20 motifs present in only one cluster and 1 motif present in 11 clusters and no motif present in more than 12 clusters. **c** Distribution of the M001 and M003 motifs relative to the TSS. **d** PLM motifs corresponding to known TF binding sites and their presence in clusters. In green, clusters mainly down-regulated; in red, clusters mainly up-regulated

To identify regulatory elements associated with flowering in mature leaves, we performed an *ab initio* search of motifs amongst the promoters of coregulated genes in each cluster, using the Preferentially Located Motifs (PLM) detector algorithm [46]. We identified 192 significant motifs of 4 to 11 bases, distributed over the 24 clusters (M001 to M192, *p*-value < 0.05) (Fig. 5, Additional file 13). We then restricted our analysis to the seven-mers or longer motifs (91 motifs). Their occurrence varied from a unique motif (M055) in 11 different clusters to 20 motifs present in only one cluster. We questioned whether these motifs corresponded to previously discovered transcription factors bindings sites (TFBSs) by using the Tomtom Motif Comparison Tool [47] and two databases of functional motifs identified by protein-binding microarrays (PBMdb) [48], and by DAP-seq (DAPdb) [49]. We kept only significant motifs (E-value < 0.01; for DAP motifs, overlap value larger than 75%). Thirteen motifs were similar to known motifs targeted by 32 TFs from these databases (Fig. 5), the remaining motifs correspond to putative novel regulatory elements, whose function will require further investigation.

The 32 TF set was enriched in GO terms associated with “regulation of metabolic process” (GO:0019222; *p*-value 5.65E-29, FDR 1.11E-26), “response to hormone stimulus” (GO:0009725; *p*-value 3.86E-12, FDR 3.53E-10) and “circadian rhythm” (GO:0007623; *p*-value 8.68E-10, FDR 6.43E-08) (PlantGSEA toolkit). The M001 motif (AAAATATCT) matched to the TFBS recognized by CCA1, LHY1, RVE1, and RVE5–8 TFs and to the “Evening Element”, involved in the control of circadian-regulated genes [50] and identified, for instance, in down-regulated genes such as *SVP, PHYTOCLOCK 1* (*PCL1*) and *LONG VEGETATIVE PHASE 1* (*LOV1*). M118 (MACGYGB) is similar to the TFBS of the MYC3 and MYC4, two TFs involved in flowering [51]. Intriguingly, no TF could be associated with M003 (AAACCCTA) and M004 (AAACCCTAA), the two closely related motifs with the best PLM *p*-values (3.43E-177, 5.39E-104, respectively). Remarkably, M003 was highly similar to the (A/G/T)AACCCTA(A/G) motif, an LHP1 binding motif, related to the *telo*-box motif (AAACCCTA) and recognized by REPEAT BINDING PROTEIN1 (TRB1) [52], and to a lesser extent, to the tertiary motif of TOE1 (AACCTTAA), a TF belonging to the AP2/EREBP superfamily (E-value 0.54 using the PBMdb). Both LHP1 and TOE1 are known to repress flowering, LHP1 being a component of PRC1 complex [53, 54] and TOE1 inhibiting the CO activity in the *FT* activation [55]. A majority of these 32 TFs (56%) were differentially regulated during the process, implying functional preferences of the identified motifs.

**Fig. 6:**
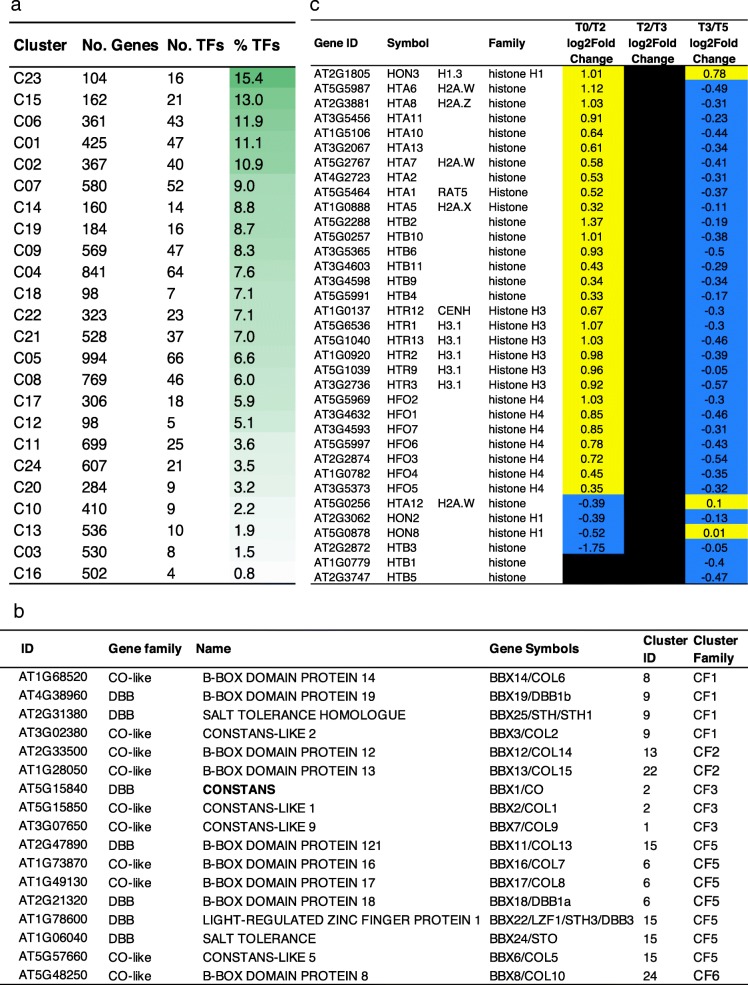
TFs and chromatin-associated genes differentially expressed during the floral transition. **a** TF Distribution in the clusters. **b** B-Box containing TFs. **c** Histone-related genes

We thus further analyzed the expression of TFs by using the PlantTFDB [56], and identified 648 differentially expressed TFs, belonging to 51 TF families (Additional file 14). *FT, TSF* and *BFT* were co-regulated with 7 other TFs (among which, *NAC3*, *NLP3*, *WOX2*, and ASG4) (C18) and *CO* and *COL1* with 38 other TFs (C02), enlarging regulatory networks. The transiently-up C23 cluster from CF1 had the highest percentage of TFs among its DEGs, in agreement with the largest transcriptional switch occurring at T0/T2 (Fig. 6a). For some TF families, a large proportion of the members were differentially expressed, suggesting important roles of these families in the response to the SD-LD shift. One of these family, the family of BBX proteins, comprises the DBB (double B-box (BBX) zinc finger protein subfamily and the CO-like subfamily [57, 58] (mainly down-regulated; 10 out of the 17 DEGs) (Fig. 6b). The BBX family comprises regulators involved in the circadian clock, photomorphogenesis, flowering time, flower development, or stress responses, such as the flowering activator COL5 [59], BBX2/COL1 a circadian clock regulator [57] or the flowering repressor COL9 [60]. These data indicate putative synergistic and antagonistic roles among the BXX family for the SD-LD switch. Thirty-nine other TFs in C2, among which 5 unknown TFs may be putative novel candidates for flowering time control in the mature leaf. The expression of the NF-Y (Nuclear Factor Y) family was also largely altered. The conserved NF-Y complexes are composed of NF-YA, NF-YB and NF-YC subunits and involved in development, stress response and flowering [61–64]. Here, the NF-YA subunits were mainly up-regulated (8 out 9 NF-YA in CF3 and CF4, the up-high and up-low cluster families) compared to the other subunits (2 NF-YB, 4 NF-YC), and some subunits were coregulated (such as NF-YA4/NF-YB2/NF-YC2 in CF3, or NF-YA8/NF-YA9/NF-YB3 in CF4), suggesting specific and dynamic compositions of NF-Y complexes. The three members of the small WHIRLY (WHY) family of single-stranded DNA binding proteins were also mainly down-regulated (C10, C13). WHIRLY1 was proposed to be involved in the gene regulation and chloroplast-to-nucleus retrograde signaling in response to redox processes occurring during light adaptation [65, 66]. While WHIRLY1 has a dual chloroplastic/nuclear localization, WHY2 and WHY3 are targeted to mitochondria and chloroplast, respectively. These data suggest a novel chloroplast-to-nucleus signaling in flowering time control.

Among FLOR-ID, a functionally related subset of 64 TFs, corresponding to the differentially expressed TFs of the database (64 out of 143) (Additional file 15) was analyzed using the TF2Network tool [67] to decipher gene regulatory networks in the mature leaf. We identified 66 specific candidate regulators, by comparison with the subset corresponding to the FLOR-ID TFs, which were not differentially expressed (data not shown). The best-ranked regulators were HYH, ABF1, TCP21, ABI5, MYC2 and HY5 (Additional file 16: Figure S7), suggesting candidate regulators of the floral transition in mature leaf. The identification of ABI5 and ABF1 as candidate regulators, which are TFs involved in ABA responses, was in agreement with the high percentage of ABA-related genes differentially expressed in mature leaf during the floral transition (Fig. 4c).

Finally, since chromatin is a key transcriptional regulatory level, we searched for key chromatin-associated genes (CAGs) involved in flowering. We identified 90 DE-CAGs, 91% being differentially expressed at T0/T2, with a bias towards up-regulated genes (Additional files 17 and 18). We noticed that a large proportion (39%) of the DE-CAGs encoded histone variants with, for instance, the *H1.3* variant (HON3), associated with stress response, 5 *H3.1* variants, which are incorporated in a replication-dependent manner in agreement with the endoreduplication events or 10 *H2A* variants (Fig. 6c). Among the genes involved in histone post-translational modifications, such as genes encoding SDG4/ASH1-RELATED 3 and SDG13/SUVR1 histone methyltransferases, were up-regulated, whereas histone deacetylases were only weakly differentially expressed, except HDA2, which was down-regulated and associated with the floral transition for the first time here. Consistently with modifications in DNA methylation accompanying early floral transition events, we observed the expression changes of *MET1*, *CMT3* but also of *DEMETER-LIKE2* (*DML2*), encoding a DNA glycosylase involved in active DNA demethylation, in the mature leaves. Thus, the results suggest a rapid modification of the epigenome, concomitant with the changed TF profiles, which further endorses a dramatic reprogramming in the leaf genome, reminiscent of another developmental switch [38].

### A set of lncTUs is differentially regulated during the floral transition

**Fig. 7:**
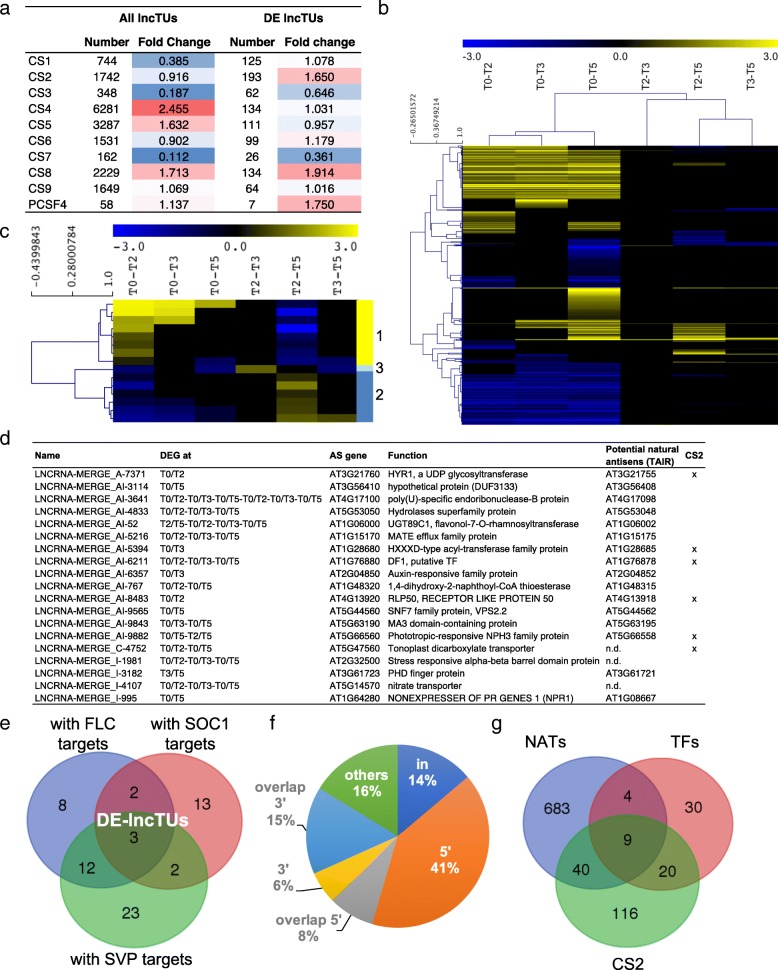
Analyses of lncRNAs during the photoperiodic inductive switch. **a** Heat maps of the lncTU distribution in the nine chromatin states and the PCFS4 target regions. The fold change was calculated based on the ratio between observed and randomly distributed lncRNAs. **b** Hierarchical clustering of the DE-lncTUs. **c** Hierarchical clustering of the dynamic DE-lncTUs. **d** DE-lncTUs in NAT orientation with genes differentially expressed at the same time point and corresponding potential NATs, as annotated in TAIR. **e** Venn diagram between the different DE-lncTU sets with FLC, SOC1 or SVP binding sites in their vicinities. **f** Distribution of the TFBSs of FLC, SOC1 or SVP inside (in), at the 5′ or 3′ end, overlapping on the 3′ or 5′ end of the lncTUs. **g** Venn diagram between DE-lncTUs in CS2, DE-lncTUs with SVP, FLC or SOC1 binding sites (TFs) in their vicinities and DE-lncTUs forming NAT couples

To identify lncTUs with putative regulatory function, we firstly questioned whether the 14,621 lncTUs regions of our dataset and the 531 DE-lncTUs were associated with specific genome topographical characteristics, especially the nine chromatin states (CS) [68] (Fig. 7a). Consistently, lncTUs were globally preferentially associated with CS4 that corresponds predominantly to distal promoter regions and non-coding intergenic regions. CS4 has high levels of H3K27me3 and reduced levels of active histone marks. To a lesser extent, but still significantly, lncTUs are prevalent in CS5, the Polycomb-regulated CS also enriched in high H3K27me3 levels, and in CS8, the AT-rich heterochromatin, but not with the constitutive heterochromatin (CS9). Whereas the fold changes for lncTUs and DE-lncTUs were quite similar in CS8, a large discrepancy was observed between the two sets in the bivalent chromatin state, CS2. We observed a low number of DE-lncTUs in regions targeted by PCF11-SIMILAR PROTEIN 4 (PCFS4), a key factor involved in flowering time and acting on the *FCA* alternative processing [69] (Fig. 7a). These results suggest the existence of sets of lncTUs associated with specific chromatin states and with specific regulatory activities during the floral transition, such as the CS2 subset of the 193 DE-lncTUs or the PCSF4 subset.

A hierarchical clustering analysis revealed that the majority of the 531 DE-lncTUs were either up- or down-regulated, whereas only 15 DE-lncTUs changed dynamically (Fig. 7b, c). This indicates that most of the of DE-lncTUs were mainly specific for either the vegetative or the reproductive phase, while fewer specific ones are involved in the transition event only.

To find out if lncTUs can change the expression of genes in *cis* we examined the expression of both the lncTUs and their neighboring genes. No correlation between expression and structure could be detected at the genome level. We then investigated whether lncTUs may have putative NAT regulatory function by analyzing lncTUs with overlapping genes. The *FLORE* NAT, for example, represses *CDF5* located in antisense but also *CDF1* and *CDF3* located on other chromosomes and participates to the flowering time control [24]. Whereas *CDF2* was up-regulated, *CDF3* and *CDF5* were down-regulated in our data. Putative lncTUs that we could identified in the regions of *CDF2*, *CDF3,* and *CDF5* were not differentially expressed in our experimental design, which suggests that other regulatory mechanisms control *CDF* expression. Alternatively, transient changes in the expression of the corresponding lncTUs could not be detected. In our dataset, we found that 655 lncTUs overlapped with a flanking gene and were transcribed in antisense (putative NAT lncTUs). Among them, 19 NAT lncTUs showed opposite transcriptional activity with the overlapping gene, in at least one of the 6 comparisons (Fig. 7d, Additional file 19). However, the expression dynamics of the gene and its NAT lncTU may be more complex and the two partners may have no synchronized expression. For instance, a lncTU formed a NAT couple with *MAF5 (*LNCRNA-MERGE_C-9859, named *MAF5_NAT*), encoding a floral repressor of the *FLC* clade. *MAF5* was differentially regulated during the floral transition and *MAF5_NAT* was strongly down-regulated before the up-regulation of MAF5 (Additional file 20: Figure S8). This is different from the transient up-regulation of antisense regulatory ncRNAs which represses *FLC*, thus illustrating the complexity of the flowering regulation mechanism.

Finally, for each DE-lncTU, we examined the presence of the TFBSs of the SVP, FLC and SOC1 flowering regulators, in a 4-kb window (3 kb upstream - 1 kb downstream) [70–74]. We identified 123 TFBSs in the vicinity of 63 DE-lncTUs, with one to seven of these binding sites mainly present in the 5′ regions, suggesting some functionality of these TFBSs regarding the expression of the DE-lncTUs and putative roles of these TFs in lncTU regulation (Fig. 7e, f, Additional file 21). Furthermore, some of the DE-lncTUs with TFBSs were located in CS2 and/or involved in NAT couples. Based on these criteria (expression profile, presence of TFBS, location in specific chromatin state, NAT couple), the identified DE-lncTUs represent interesting candidates whose regulatory function in flowering will require further investigation.

## Discussion

### Complex molecular processes in mature leaves during the floral transition

In sensing floral inductive stimuli, leaves produce the florigen signal that switches the SAM from the vegetative to reproductive phase. Key genes involved in producing the florigen have been identified, but the global molecular events in leaves during the floral transition remain poorly described. By focusing on mature rosette leaf whose growth was completed, our study completes analyses and highlights molecular events of the floral transition in this organ. Based on differential gene expression profiles, we showed that the floral transition induced by the SD-LD switch is accompanied by re-organization of photosynthetic capacity, protein synthesis, cellular metabolism, hormonal action, stress response and cell cycle regulation, with an intricate interplay between the light regime, the circadian clock and the floral transition. Our data highlight the complex role of mature leaves in the floral transition.

LD stimulates increases in leaf sucrose level as part of the florigenic signal [75–77]. However, other metabolites are also involved in the floral transition: carbon, phosphorous, nitrogen or sulphur can impact this process [4, 78]. Genes associated with flavonoids and glucosinolates contents were differentially regulated during the photoperiodic switch, whereas these secondary metabolites are usually associated with stress responses [79]. These data support a recent study showing that the flowering regulator *FLC* is present in a QTL interval associated with glucosinolate contents in the brassicaceae species, *Aethionema arabicum* [80]. The switch occurring in the SAM appears to require massive metabolic and physiologic reprogramming events in the leaf to further explore.

### Highlight of new regulatory candidates in flowering control

Dynamics changes were reported in the transcriptional profiles of genes but also lncRNAs, highlighting the potential regulatory functions of some of them in the floral transition. Our atlas of lncTUs, with putative regulatory functions based, for instance, on their location in bivalent chromatin states or in antisense with protein-coding genes, provides promising resource for new actors in the genome regulation during the floral transition.

The analysis of the gene clusters allowed the extraction of specific putative regulatory motifs. Some of these DNA elements corresponded to binding sites of differentially expressed TFs, suggesting functionality in the transcriptional regulation of the floral transition. In parallel, a large set of differentially expressed TFs involved in various processes was identified, consistently with the molecular processes highlighted with the GO analysis. Changes in transcription of a set of FMI-FD genes, whose action is crucial in meristems, suggest other levels of gene regulation during the floral transition.

By focusing on FLOR-ID TFs, we highlighted small gene regulatory networks and potentially, new players in the floral transition in leaf, such as ABI5, ABF1, or TCP21. TCP21 is involved in the circadian clock regulation and controls *CCA1* expression [81]. The identification of TCP21 as candidate regulator here is in agreement with the large proportion (84.2%) of circadian clock–associated genes differentially expressed, during the SD-LD switch (Fig. 4b). ABI5 was reported as a floral repressor [82], which is consistent with its down-regulation here during the SD-LD switch and its putative interactions with the downstream deregulated TFs. ABA is involved in the control of flowering time with opposite effects according to environmental conditions [83–85]. For instance, ABA was shown to promote flowering time in a LD-dependent manner and in response to water resource availability, by modulating *GIGANTEA* (*GI*) activity on *FT* and *TSF* [84, 85]. However, the down-regulation of *GI* here suggests that the ABA-dependent promotion of flowering may not be involved in the SD-LD transition, but a loss of repression mediated by ABI5 may possibly occur. The hormonal contribution of the GA-dependent promoting pathway [10] may also play a role. Most of the hormone pathways being affected during the switch, albeit to different degrees, performing hormone dosages may help to untangle their contribution to flowering time.

### Endoreduplication events accompanying the floral transition in leaves

The acceleration of the cell division was reported in the SAM during the floral transition in *A. thaliana* [29, 86]. Here, we report that endocycles escort the floral transition in leaves. Thus, the floral induction is accompanied by modulations of the cell cycle in both leaves and meristems, but with differences in cell cycle exits according to the organs, endocycle or mitosis, respectively. Our result is supported by the changes in expression of key cell cycle phase markers, such as *CYCA2;3*, but also histone variants and endocycle-related genes. Consistently with a loss of function stimulating endocycles [87], *CYCD3* genes were down-regulated in our experiments. The spindle assembly checkpoint (SAC) genes were also shown to impact both the floral transition and the timing of the endocycle onset [88]. Here, only BUBR1/MAD3 from the SAC family was slightly down-regulated, but its function in the Arabidopsis mitotic checkpoint control remain poorly documented.

Hormone signaling pathways participate to the control of the mitotic-to-endocycle transition: the endocycle repression is induced by high auxin contents [39, 89]. Consistently, we observed alterations of the auxin pathways during the photoperiodic inductive switch. The SUMO E3 ligase HPY2, described as an endocycle repressor, which may link auxin signaling and cell cycle program [89, 90], as well as two other negative regulators of endocycles, were up-regulated at the T0/T2 transition, suggesting that the entry into the endocycle program in the mature leaves may result from a fine dosage between the different controlling pathways.

Previous studies showed that the increase in light intensity [91] and UV-B radiation [92] are associated with changes in ploidy levels. A proposed hypothesis is that the ploidy dynamics might be an adaptive response to damage possibly induced by solar radiation. Finally, we could also speculate that the increase in ploidy level in mature leaves during the floral transition may contribute to an increase in energy production required for the developmental switch, an increase in metabolites and endogenous signaling molecules, or a modulation of transcription thresholds.

## Conclusions

Our detailed study provides a novel molecular framework to further question the roles of new putative regulators in leaves during the floral transition, such as new putative lncRNAs, whose polyadenylation status will require further confirmation. Furthermore, it points at the relationship between flowering and endoreduplication and at the complex interplay between several plant hormones, which open new perspectives.

## Methods

### Plant materials and growth conditions

All *Arabidopsis thaliana* lines were in the Col-0 background. Seeds of the *pAP1::GUS* transgenic line were kindly provided by Prof. Dr. G. Angenent (unpublished material). The *AP1* promoter fragment was fused to a *GUS-GFP* cassette as described in [93], using the binary pBGWFS7 vector from VIB [94]. Plants were grown in growth chamber under SD (8 h light/16 h dark) or LD (16 h light/8 h dark) conditions. White fluorescent light was used. The photosynthetic photon flux density was 120 μmol m^− 2^ s^− 1^ in SD and LD. In SD, the temperature was 21 °C during the light and 18 °C during the dark period, and the humidity (65%) remained constant. In LD, the temperature (21 °C) and the humidity (70%) remained constant, 21 °C and 70%, respectively. Plants were cultured for 3, 4 or 5 weeks in soil, in individual pot. The transfer was done at the end of SD light, preceding the LD dark period. Flowering time indicators were recorded as previously described [53](Additional file 1: Figure S1a). The percentages of cauline leaf relatively to the total leaf number (CL%) quantifies the relationship between bolting and floral transition events [95]. For plants grown 3, 4 and 5 weeks in SD and transferred in LD it similar to the CL% in continuous LD (17.6%), and higher compared to continuous SD (11.9%) [96]. This preliminary assay showed that the SD-LD switch mimicked LD growth conditions. For a good compromise between time and material quantity, analyses were then pursued on plants grown for 4 weeks in SD.

For RNA extraction, plants were collected at Zeitgeber time 7 (ZT7) in SD, and ZT15 in LD, ZT0 marking the transition from dark to light. For leaf growth analysis, individual leaves were harvested at different time points, flattened on white paper and then digitally scanned. Leaf areas (blade and petiole) were calculated from the binary images using ImageJ software (http://rsb.info.nih.gov/ij/). Leaves from 10 to 15 plants were analyzed.

### Ploidy analysis

Leaves 1 to 4 were harvested, chopped with a razor blade in 800 μl of Galbraith buffer, filtered over a 30 μm mesh, and 150 μl of a propidium iodide solution (100 μg/ml) was added [97]. The quantification of the nuclear DNA content was performed on a CyFlow® cytometer using the FloMax® software (Sysmex Partec, France) as described [98]. The endoreduplication index was calculated by using the formula: EI = 0x(% of 2C) + 1x(% of 4C) + 2x(% of 8C) + 3x(% of 16C) + 4x(% of 32C).

### Expression analysis

Total RNAs were prepared from rosette material, treated and reverse transcribed, as previously described [99]. Quantitative real-time PCR was performed on a BioRad CFX96 apparatus using the SYBR green Master Mix (BioRad) following manufacturer’s instructions. *UBIQUITIN10* was used as reference gene. Primers are listed in Additional file 22. For GUS histochemical staining, plants were collected in the staining solution (1 mM X-Gluc (5-bromo-4-chloro-3-indolyl-ß-D-glucuronide), 0.1 M sodium phosphate buffer, pH 7.0, 2 mM potassium ferrocyanide, 2 mM potassium ferricyanide, and 0.5% Triton X-100), infiltrated under vacuum 3 times, for 5 min each, and incubated at 37 °C overnight. Samples were then washed in 70% ethanol and observed under a light microscope.

### RNA extraction, library preparation and sequencing

Total RNA was extracted with the Plant RNeasy Mini kit (QIAGEN). 10 μg of RNA was treated with TURBO DNA-free kit (Ambion Ref. AM1907) and cleaned-up from enzymatic reactions with RNeasy MinElute Cleanup Kit (QIAGEN Ref. 74,204), following the manufacture instructions. RNA integrity and concentration were analyzed with the Agilent 2100 Bioanalyzer and the Agilent RNA 6000 Nano Kit (Ref. 5067–1511). For one replicate, leaves 3–4 were dissected from 20 plants and pooled. Three independent replicates were performed for each time point. Strand specific sequencing libraries were prepared from polyA RNAs using the Illumina Tru-Seq stranded RNA sample preparation v2 kit. Four libraries were multiplexed per lane and paired-end (PE) sequenced on an Illumina HiSeq 2000. Over 40 millions of 150 bp reads were generated per sample. All steps of the experiment, from growth conditions to bioinformatic analyses, were recorded in CATdb database [100] (http://tools.ips2.u-psud.fr/CATdb/) Project ID NGS2015_01_Transition according to the international standard MINSEQE minimum information about a high-throughput sequencing experiment.

### RNA-Seq data analysis

RNA-Seq samples were processed using the following pipeline: the read pre-processing criteria included trimming library adapters and performing quality control checks using FastQC. The raw data (fastq) were trimmed using the FastX toolkit (Phred Quality Score > 20, read length > 30 bases). The Bowtie 2 mapper [101] was used to align reads against the *A. thaliana* TAIR 10 transcriptome. On average, 99% passed the quality filter and were uniquely mapped to the TAIR 10 reference genome. We extracted 33,602 genes from TAIR10 version database [102] with one isoform per gene corresponding to the representative gene model (longest coding sequence) given by TAIR10. The abundance of each gene was calculated by a local script, which parses SAM files and counts only paired-end reads for which both reads map unambiguously one gene, and by removing multi-hits. According to these rules, around 96% of PE reads were associated with a gene, 2% PE reads unmapped and 2% of PE reads with multi-hits were removed.

For differential expression analysis, we discarded genes, which did not have at least 1 read after a count per million (CPM) normalization, in at least one half of the samples. The library sizes were normalized using the TMM method. The count distribution was modelled with a negative binomial Generalized Linear Model (GLM) where the harvest date was considered. Dispersion was estimated by the edgeR method [103] in the statistical software ‘R’ (R Core team, 2015). The *p*-values were adjusted by the Benjamini-Hochberg procedure to control FDR. A gene was differentially expressed when its adjusted p-value was lower than 0.05.

### Analysis of lncRNAs

We gathered a non-redundant dataset (IJPB_lncDB) of 14,621 putative lncRNA sequences from published lncRNAs datasets [26, 104–106]. Redundant information was removed. Datasets were organized into three subsets according to strand information (+, −, Not Available (NA)). For each subset, we merged overlapping or “book-ended” lncRNA in a single transcription unit (lncTU). Three FASTA files of lncTUs were established: one with 5055 TUs on the positive strand, another one with 4851 putative TUs on the negative strand, and the last one with 4715 TUs, without strand information. All reads were mapped against the IJPB_lncDB using the Bowtie 2 mapper [107] using the same count criteria. Whereas 96% of the paired-end reads mapped to the TAIR10 genome as expected, the mean mapping percentage to the lncRNA dataset was 0.78%. To establish the differentially expressed lncTUs, we used the GLM of edgeR, without or with filter using either the Bonferroni or Benjamini-Hochberg (BH) test corrections. All lncTUs differentially expressed identified using Bonferroni test were present in the list of DE lncTUs identified using BH test. We further analyzed BH DE lncTUs. No bias was observed for the distribution of the DE-lncRNAs on the two strands.

To determine overlaps between lncRNA and annotated chromatin states we used the online BEDTools suite. We established intersects for all lncRNAs, the DE lncRNAs and randomly reshuffled regions of identical size to compute the fold changes between observed and randomly distributed lncRNAs. The hierarchical clustering analysis was performed using the Multiexperiment Viewer tool (MeV 4_8) with the average linkage method, gene leaf order optimization and Pearson correlations.

### Model for the co-expression analysis

Co-expression analysis was carried out on differentially expressed transcripts and lncRNAs using the R package coseq [108] (https://bioconductor.org/packages/devel/bioc/vignettes/coseq/inst/doc/coseq.html). We ran two clustering methods (*K*-means algorithm and Gaussian mixture models) for two different count data transformation functions (the centred log ratio (CLR) and logCLR for *K*-means; Logit and arcsin for Gaussian mixture models). Ten technical replicates were performed for each combination of method/transformation to prevent initialization problems. We computed 30 models from *K* = 10 to *K* = 40 (*K* = number of clusters). For each method, the best *K* was selected via the slope heuristics approach for *K*-means methods or via the Integrated Completed Likelihood (ICL) criterion for Gaussian mixture models. The transformation function, which minimizes the within clusters variability (for *K*-means algorithm) or the ICL criterion (for Gaussian mixture models) was retained. Since the *K*-means algorithm seemed more sensitive to extreme expression data, we finally retained the Gaussian mixture model method with the arcsin transformation function and K = 24. This method provided a more homogeneous number of transcripts per cluster.

For each cluster, a Singular Enrichment Analysis (SEA) of GO terms (AgriGO v2.0) [109] was performed (Fisher test with a FDR cut-off at 0.01 and a minimum number of mapping entries of 10), using a customized reference corresponding to expressed genes during the time course experiment. An heatmap comparing the results of individual cluster’s SEA were obtained using the SEACOMPARE program (AgriGO v2.0).

### Bioinformatics analysis

We extracted 413 genes (FLGs) from FLOR-ID [16], comprising the 306 core flowering time genes, genes involved in flower meristem identity and flower development (FMI-FD) and pending annotated flowering time genes. For the analysis of the TFs we used the PlantTFDB 4.0 (http://planttfdb.cbi.pku.edu.cn/). Chromatin-associated genes were described in [38]. Hierarchical clustering was performed using the Multiple Experiment Viewer tool (MeV) with the Pearson correlation metric and average linkage clustering as linkage method [110]. We performed functional annotation and classification using the **“**AgriGO**”** Gene Ontology tool [109] and the Classification SuperViewer Tool from BAR [111]. For each cluster, we extracted the biological process (BP) GO terms with the best FDR and the best specialized and enriched “child” GO terms (Additional file 23). Venn diagrams were generated using the online tool provided by T. Hulsen (http://bioinformatics.psb.ugent.be/webtools/Venn/).

### Motif detection

The “Preferentially Located Motifs” algorithm is based on the over-representation of a motif around the Transcription Start Site (TSS), region − 300 from TSS to 5’UTR, compared to its distribution in the region of − 1000 to − 300 (learning region) before the TSS [46]. We also explored a list of 419 motifs merged from PLACE [112] and AGRIS (http://agris-knowledgebase.org/AtcisDB/bindingsites.html) to find enrichment (*p*-value < 0.05) around the TSS compared to all Arabidopsis genome (Additional file 23).

### Accession number

The accession number into the international GEO repository is GSE116123.

## Additional files

Additional file 1: **Figure S1.** Characterization of the SD-LD switch. (a) Flowering time according to the number of weeks in SD. Rosette and cauline leaves were recorded on plants when the first flowers appeared. The bolting time was quantified when the stem was 0.5 cm high from sowing. Three biological replicates were performed with 12 plants, each. (b) Expression of the *AP1::GUS* reporter gene in the apical shoots of plants grown 4 weeks in SD, and then transferred to LD. Day after transfer (dat). (PDF 772 kb)

Additional file 2: **Figure S2.** Growth of rosette leaves in response to SD or a SD-LD switch. (a) Measurements of the total rosette leaf areas. (b) Area measurement of the first six leaves. Col-0 plants were grown in SD for 4 weeks, then kept in SD (continuous line) or transferred in LD (dash line). Two biological replicates were performed with 10 plants, each. Experimental values are mean ± SEM. (PDF 70 kb)

Additional file 3: **Figure S3.** RNA-Seq experiments and expressed gene distributions. (a) Library sizes of the biological replicates. (b) Read mapping for the different time points and biological replicates. (c) Distribution of the expressed genes in the genomes. (d) Differentially expressed genes (DEGs) and Venn diagram with the three main comparisons. (e) Distribution of the expressed genes in the main gene classes. (PDF 194 kb)

Additional file 4: **Figure S4.** Analysis of lncRNAs. (a) Resources used to construct the lncTU dataset. (b) lncTUs differentially expressed for each comparison using the Benjamini-Hochberg (BH) test. (PDF 51 kb)

Additional file 5: List of lncTUs. (XLSX 639 kb)

Additional file 6: List of genes per cluster. (XLSX 187 kb)

Additional file 7: Best enriched biological process (BP) GO terms for the 24 clusters. The best BP GO term was extracted with its FDR from each SEA analysis. (XLSX 11 kb)

Additional file 8: **Figure S5.** Differentially expressed endoreduplication-related genes extracted from the ThaleMine database. Log2 (Fold-Change) is reported. In black, non-statistically significant fold change values. (PDF 44 kb)

Additional file 9: Distributions of differentially expressed genes of the FLOR-ID database in the clusters. (XLSX 28 kb)

Additional file 10: Hormone-related genes supported by genetic evidence in clusters. (XLSX 22 kb)

Additional file 11: **Figure S6.** Floral transition and hormone pathways. (a) Distribution of AHD genes in clusters. (b) Genes associated with gibberellin metabolism and responses. (PDF 56 kb)

Additional file 12: Hormone-related genes involved in several hormonal pathways in the clusters. (XLSX 10 kb)

Additional file 13: Motifs identified with the PLM algorithm. (XLSX 87 kb)

Additional file 14: Distribution of the TFs among the clusters. (XLSX 38 kb)

Additional file 15: TFs from the FLOR-ID. (XLSX 12 kb)

Additional file 16: **Figure S7.** Screenshots of the TF2network user interface using the 64 TFs differentially regulated and belonging to FLOR-ID. The three Cytoscape panels show the gene regulatory networks with the first 5 best-ranked regulators (blue diamonds). Green diamonds represent TFs and green circles, non-TF genes, according to the TF2Network interface annotations. The dashed arrows indicated PWM motifs for the corresponding regulators. The blue lines indicate protein-protein interactions. (PDF 524 kb)

Additional file 17: Differentially expressed genes associated with chromatin biology. (XLSX 19 kb)

Additional file 18: Distribution of differentially expressed genes associated with chromatin biology. CR: chromatin remodeling. (XLSX 9 kb)

Additional file 19: Expression of lncTUs and their NATs. (XLSX 25 kb)

Additional file 20: **Figure S8.** Expression of MAF5 and its antisense lncTU located in the 3′ end of *MAF5* region at T0, T2 and T3. (a) Fold changes. (b) Browser snapshot showing the expression profiles. BR1: biological replicate number 1. (PDF 77 kb)

Additional file 21: Distribution of the TFBFs in the regions of the lncTUs. (XLSX 21 kb)

Additional file 22: List of oligonucleotides. (XLSX 8 kb)

Additional file 23: Information in PLACE and AGRIS databases on the identified motifs. (XLSX 38 kb)

## Abbreviations

DEG: Differentially expressed gene
FT: Transcription factor
GO: Gene ontology
LD: Long days
lnc: Long non-coding
SD: Short days
TU: Transcription unit
ZT: Zeitgeber
