# Supplementary material for: Extensive nuclear reprogramming and endoreduplication in mature leaf during floral induction
Source: BMC Plant Biol. 2019 Apr 11;19:135. doi: 10.1186/s12870-019-1738-6 (PMC6458719; doi:10.1186/s12870-019-1738-6)

a

| Number of weeks in SD | 3 | 4 | 5 |
|---|---|---|---|
| *Flowering parameters* | | | |
| Rosette leaves | 26.3 ± 0.1 | 33.6 ± 1.3 | 41.2 ± 1.5 |
| Cauline leaves | 5.8 ± 0.1 | 6.5 ± 0.1 | 8.8 ± 0.6 |
| Total leaves | 32.1 ± 0.2 | 40.2 ± 1.5 | 50.1 ± 1.6 |
| % cauline leaves | 18.1 ± 1.1 | 16.3 ± 1.6 | 17.7 ± 1.2 |
| No. days to bolt from sowing | 35 ± 1 | 41 ± 1 | 47 ± 1 |
| No. days to bolt from transfer | 14 ± 1 | 13±1 | 12 ± 1 |
| *Light conditions* | | | |
| Light hours in SD | 168 | 224 | 280 |
| Light hours in LD | 240 | 224 | 176 |
| Total light hours | 408 | 432 | 456 |

b

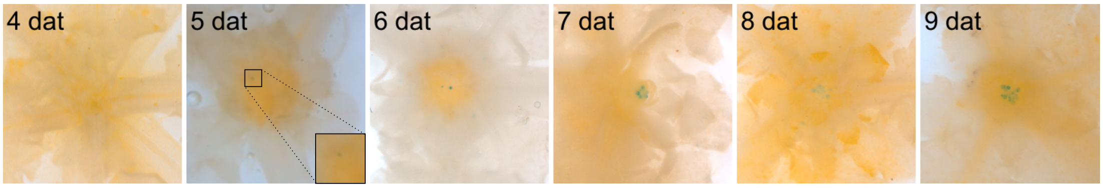

Supplement: Supplementary file 1 — Figure S1. Characterization of the SD-LD switch. (a) Flowering time according to the number of weeks in SD. Rosette and cauline leaves were recorded on plants when the first flowers appeared. The bolting time was quantified when the stem was 0.5 cm high from sowing. Three biological replicates were performed with 12 plants, each. (b) Expression of the AP1::GUS reporter gene in the apical shoots of plants grown 4 weeks in SD, and then transferred to LD. Day after transfer (dat). (PDF 772 kb) [file 12870_2019_1738_MOESM1_ESM.pdf]
