# Supplementary figures and images for: Extensive nuclear reprogramming and endoreduplication in mature leaf during floral induction
Source: BMC Plant Biol. 2019 Apr 11;19:135. doi: 10.1186/s12870-019-1738-6 (PMC6458719; doi:10.1186/s12870-019-1738-6)

**a**

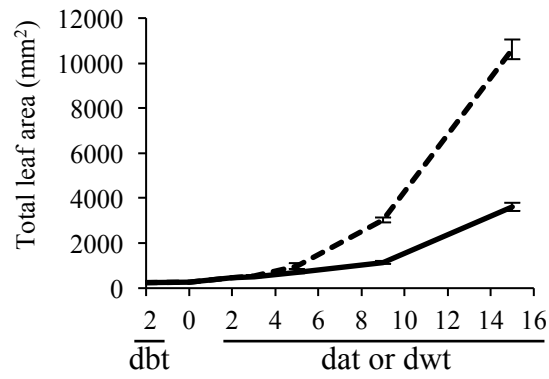

**b**

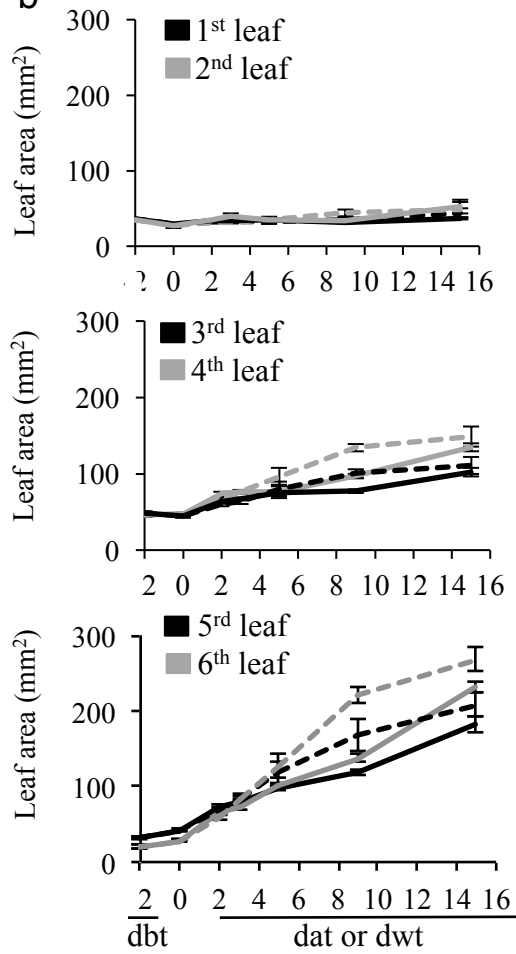

Supplement: Supplementary file 2 — Figure S2. Growth of rosette leaves in response to SD or a SD-LD switch. (a) Measurements of the total rosette leaf areas. (b) Area measurement of the first six leaves. Col-0 plants were grown in SD for 4 weeks, then kept in SD (continuous line) or transferred in LD (dash line). Two biological replicates were performed with 10 plants, each. Experimental values are mean ± SEM. (PDF 70 kb) [file 12870_2019_1738_MOESM2_ESM.pdf]

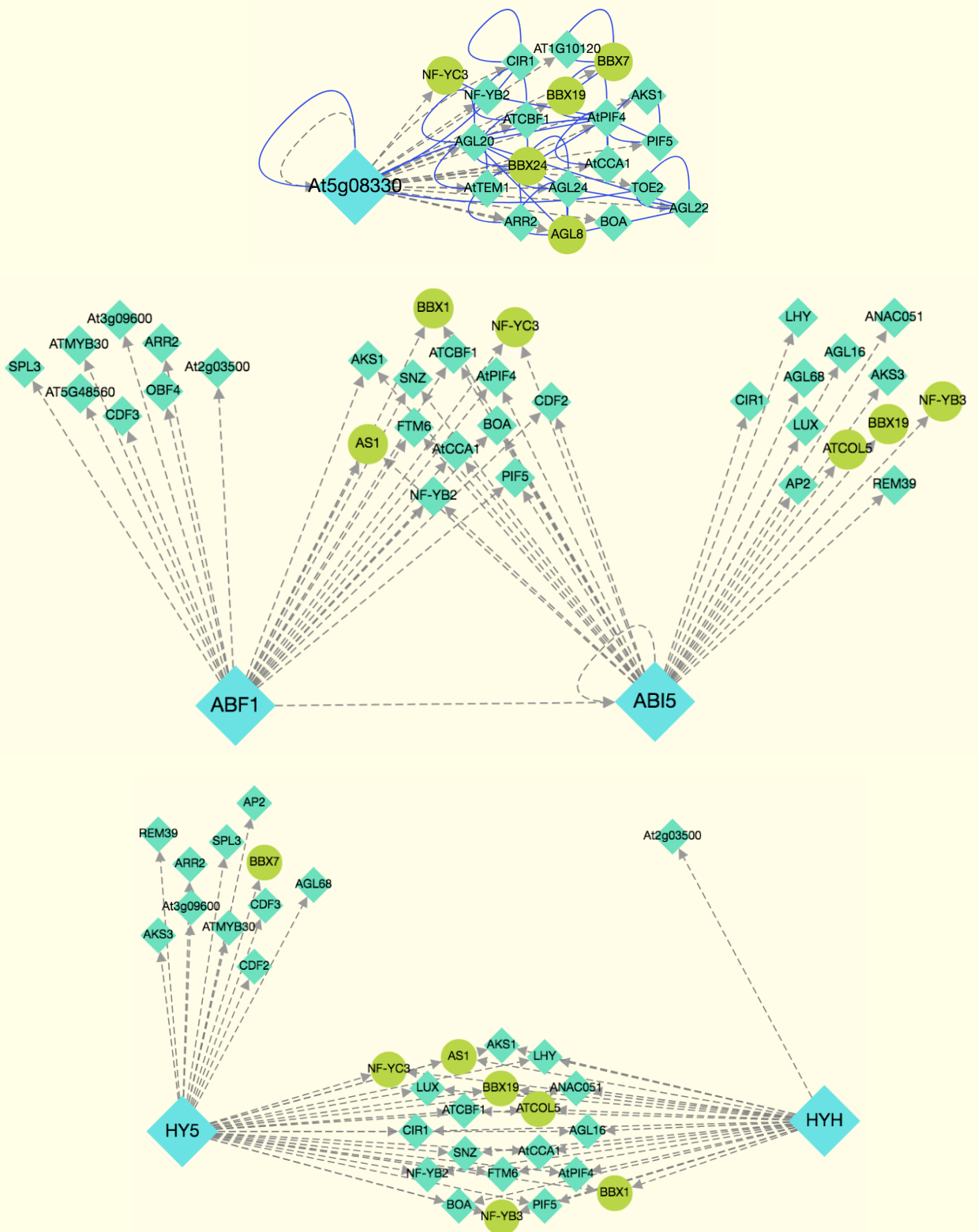

Supplement: Supplementary file 16 — Figure S7. Screenshots of the TF2network user interface using the 64 TFs differentially regulated and belonging to FLOR-ID. The three Cytoscape panels show the gene regulatory networks with the first 5 best-ranked regulators (blue diamonds). Green diamonds represent TFs and green circles, non-TF genes, according to the TF2Network interface annotations. The dashed arrows indicated PWM motifs for the corresponding regulators. The blue lines indicate protein-protein interactions. (PDF 524 kb) [file 12870_2019_1738_MOESM16_ESM.pdf]
