# Supplementary material for: Extensive nuclear reprogramming and endoreduplication in mature leaf during floral induction
Source: BMC Plant Biol. 2019 Apr 11;19:135. doi: 10.1186/s12870-019-1738-6 (PMC6458719; doi:10.1186/s12870-019-1738-6)
Supplement: Supplementary file 3 — Figure S3. RNA-Seq experiments and expressed gene distributions. (a) Library sizes of the biological replicates. (b) Read mapping for the different time points and biological replicates. (c) Distribution of the expressed genes in the genomes. (d) Differentially expressed genes (DEGs) and Venn diagram with the three main comparisons. (e) Distribution of the expressed genes in the main gene classes. (PDF 194 kb) [file 12870_2019_1738_MOESM3_ESM.pdf]

a

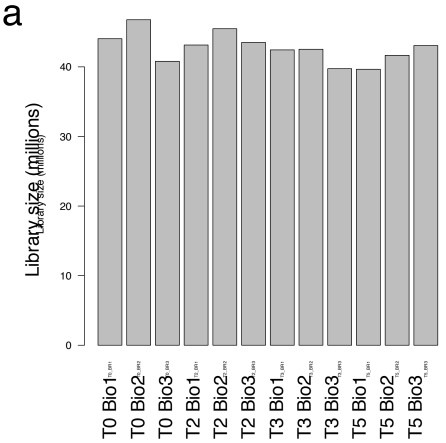

b

		Bowtie2 results		*Sam2counts*		
**Time point**	**Biological repeat**	**Total paired reads**	**Overall alignment rate**	**Total multi-hits read removed**	**Total multi-hits pair removed**	**Total pair counted (properly paired on same ref. and reversed strand)**
T0	BR01	45 542 220	98.97%	1 554 854	529 044	44 044 212
	BR02	48 165 370	99.23%	1 676 322	559 347	46 770 727
	BR03	42 134 256	99.20%	1 517 241	533 998	40 798 729
T2	BR01	44 506 108	99.03%	1 540 091	605 070	43 139 269
	BR02	47 126 174	99.08%	1 712 362	692 819	45 480 238
	BR03	45 078 232	99.09%	1 556 387	620 648	43 504 758
T3	BR01	44 073 307	99.12%	1 584 790	651 057	42 437 368
	BR02	44 189 464	98.78%	1 604 125	651 570	42 527 353
	BR03	41 052 667	99.15%	1 419 324	571 364	39 737 795
T5	BR01	41 046 783	99.07%	1 428 504	581 646	39 655 480
	BR02	43 207 137	99.04%	1 520 445	628 679	41 657 733
	BR03	44 543 187	99.08%	1 536 824	635 666	43 059 175

c

**Time point**	**Nuclear gene No.**	**Mitochondrial gene No.**	**Chloroplastic gene No.**
T0	19 831	36	73
T2	19 644	34	72
T3	19 707	37	76
T5	19 780	40	71

d

	**DEG No.**		
**Comparison**	**All**	**Up**	**Down**
T0/T2	6 582	3 322	3 260
T2/T3	1 012	470	542
T3/T5	1 673	703	970

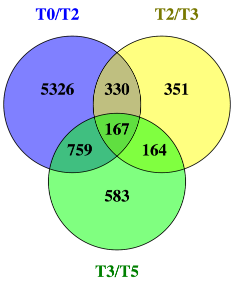

e

**Genes**	**No. in the genome (TAIR10)**	**All expressed genes**	**% in the class**	**Expressed in T0**	**% in the class**	**Expressed in T2**	**% in the class**	**Expressed in T3**	**% in the class**	**Expressed in T5**	**% in the class**
Protein coding	27416	19515	71.2	19118	69.7	19055	69.5	19103	69.7	19189	70
TE	3903	206	5.3	188	4.8	179	4.6	185	4.7	186	4.8
Pseudogenes	924	200	21.6	182	19.7	172	18.6	184	19.9	175	18.9
tRNA	689	7	1	4	0.6	4	0.6	5	0.7	5	0.7
ncRNA	394	297	75.4	288	73.1	286	72.6	289	73.4	287	72.8
miRNA	177	28	15.8	24	13.6	25	14.1	26	14.7	24	13.6
snoRNA	71	24	33.8	21	29.6	23	32.4	21	29.6	18	25.4
rRNA	15	0	0	0	0	0	0	0	0	0	0
snRNA	13	7	53.8	6	46.2	6	46.2	7	53.8	7	53.8
Total	33602	20284		19831		19750		19820		19891	
