# Supplementary material for: Extensive nuclear reprogramming and endoreduplication in mature leaf during floral induction
Source: BMC Plant Biol. 2019 Apr 11;19:135. doi: 10.1186/s12870-019-1738-6 (PMC6458719; doi:10.1186/s12870-019-1738-6)
Supplement: Supplementary file 4 — Figure S4. Analysis of lncRNAs. (a) Resources used to construct the lncTU dataset. (b) lncTUs differentially expressed for each comparison using the Benjamini-Hochberg (BH) test. (PDF 51 kb) [file 12870_2019_1738_MOESM4_ESM.pdf]

a

Data set	Annotations	Reference
36	Other RNAs, TAIR9	Liu et al., 2012
469	Annotated	Di et al., 2014
995	Predicted levels 1 & 2	Di et al., 2014
278	New RNA-seq (167 in RepTAS db)	Liu et al., 2012
36	Analyse EST Unigene (14 in RepTAS db)	Liu et al., 2012
32	Analyse tiling array,(26 in RepTAS)	Liu et al., 2012
61	Analyse tiling array, (39 in RepTAS)	Liu et al., 2012
6480	New data, RepTAS	Liu et al., 2012
5049	http://www.noncode.org	NONCODEv4
4396	Predicted level 3	Di et al., 2014
98	http://tools.ips2.u-psud.fr/projects/FLAGdb++/	FLAGdb++

b

	T0/T2	T0/T3	T0/T5	T2/T3	T2/T5	T3/T5	Total
DE-lncTUs	313	291	456	5	95	28	531
