# Supplementary material for: Extensive nuclear reprogramming and endoreduplication in mature leaf during floral induction
Source: BMC Plant Biol. 2019 Apr 11;19:135. doi: 10.1186/s12870-019-1738-6 (PMC6458719; doi:10.1186/s12870-019-1738-6)
Supplement: Supplementary file 8 — Figure S5. Differentially expressed endoreduplication-related genes extracted from the ThaleMine database. Log2 (Fold-Change) is reported. In black, non-statistically significant fold change values. (PDF 44 kb) [file 12870_2019_1738_MOESM8_ESM.pdf]

Gene ID	Symbol	Name	T0/T2 log2Fold Change	T2/T3 log2Fold Change	T3/T5 log2Fold Change
GO:0042023 - DNA endoreduplication					
AT1G47870	E2Fc	Homolog of E2F C	0.58		
AT3G19150	KRP6	KIP-related protein 6	0.71		
AT3G48160	DEL1	DP-E2F-like 1	0.83		
AT3G50630	KRP2	KIP-related protein 2	0.53		
AT1G15570	CYCA2;3	Cyclin A2;3	-0.79		-0.94
AT2G27960	CKS1	Cyclin-dependent kinase 1	-0.51	0.32	
AT2G42260	UVI4	UV-B-Insensitive 4	1.16		
GO:0032876 - negative regulation of endocycle					
AT3G15150	HPY2	High ploidy2	0.68		
AT2G18290	APC10	Anaphase promoting complex 10	-0.94		
AT3G48160	DEL1	DP-E2F-like 1	0.83		
AT1G33240	GTL1	GT-2-like 1	0.50		-0.37
GO:0032877 - positive regulation of endocycle					
AT3G10525	LGO	Loss of giant cells from organs	1.17	0.29	
AT3G24810	ICK3	Cyclin-dependent kinase inhibitor 3	0.87		
AT4G02980	ABP1	ER auxin binding protein 1	0.34		
AT1G55350	DEK1	Defective Kernel 1	0.33		
GO:0032875 - regulation of endoreduplication					
AT1G78770	APC6	Anaphase promoting complex 6	0.65		
AT2G18290	APC10	Anaphase promoting complex 10	-0.94		
AT1G19270	DA1	DA1	-0.95		
AT1G69380	RRG	Retarded root growth	-0.39		
AT5G11510	MYB3R-4	Myb domain protein 3r-4	0.47		-0.68
