# Supplementary material for: Extensive nuclear reprogramming and endoreduplication in mature leaf during floral induction
Source: BMC Plant Biol. 2019 Apr 11;19:135. doi: 10.1186/s12870-019-1738-6 (PMC6458719; doi:10.1186/s12870-019-1738-6)
Supplement: Supplementary file 11 — Figure S6. Floral transition and hormone pathways. (a) Distribution of AHD genes in clusters. (b) Genes associated with gibberellin metabolism and responses. (PDF 56 kb) [file 12870_2019_1738_MOESM11_ESM.pdf]

a

Cluster	AHD DEGs (%)	p-value
23	16.35	4.74E-11
14	9.38	1.27E-06
15	7.41	1.46E-04
6	4.99	5.80E-04
7	3.79	5.00E-03
4	3.33	9.65E-03
All	3.17	1.48E-20

b

Gene ID	Symbol	Name	Function	T0/T2 log2Fold Change	T2/T3 log2Fold Change	T3/T5 log2Fold Change
Giberellin-associated genes						
AT5G56300	GAMT2	GA METHYLTRANSFERASE 2	GA metabolism		0.79	
AT1G80340	GA3OX2	GA 3-OXIDASE 2	GA9 to bioactive GA4 tranformation	2.8	0.62	
AT1G30040	GA2OX2	GA 2-OXIDASE	GA4 catabolism	0.9		
AT1G66350	RGL1	RGA-like 1	GRAS TF, Negative regulator of GA responses	1.54		
AT3G03450	RGL2	RGA-like 2	GRAS TF, Negative regulator of GA responses	1.05		
AT1G74670	GASA6	GA-STIMULATED ARABIDOPSIS 6	GA-regulated family protein	0.57		-1.28
AT4G32980	ATH1	Homeobox protein	TF, regulating GA biosynthesis	0.31		
AT3G63010	GID1B	GA INSENSITIVE DWARF1A	GA receptor			0.67
AT3G05120	GID1A	GA INSENSITIVE DWARF1A	GA receptor	-0.47		
AT5G27320	GID1C	GA INSENSITIVE DWARF1C	GA receptor	-0.91		
AT1G79460	GA2/KS1	ENT-KAURENE SYNTHASE	GA biosynthesis	-1.5		0.47
AT1G15550	GA3OX1	GA 3-OXIDASE 1	GA biosynthesis	-1.15	-1.01	
AT4G25420	GA20OX1	GA 20-OXIDASE 1	GA biosynthesis	-1.24		
AT1G78440	ATGA2OX1	GA 2-beta-DIOXYGENASE	GA metabolism	-4.38		
AT1G22690		Unknown	GA-regulated family protein	-1.23		-0.87
AT5G14920	GASA14	GA-STIMULATED IN ARABIDOPSIS 14	GA-regulated family protein			-0.71
Cytokinin-associated genes						
AT5G06300	LOG7	LONELY GUY 7	cytokinin biosynthesis	0.9		1.11
AT5G11950	LOG8	LONELY GUY 8	cytokinin biosynthesis	0.67		
AT5G20040	IPT9	tRNA ISOPENTENYLTRANSFERASE 9	cytokinin biosynthesis	-0.71		
AT4G29740	CKX4	CYTOKININ OXIDASE 4	cytokinin catabolism	-0.89		
AT3G63440	CKX6	CYTOKININ OXIDASE/DEHYDROGENASE 6	cytokinin catabolism			-0.66
AT5G21482	CKX7	CYTOKININ OXIDASE 7	cytokinin catabolism	1.05		
AT1G26210	SOFL1	SOB FIVE-LIKE 1	regulator of cytokinin levels	0.7		-1.35
AT1G68870	SOFL2	SOB FIVE-LIKE 2	regulator of cytokinin levels	1.41		
