# Supplementary material for: Extensive nuclear reprogramming and endoreduplication in mature leaf during floral induction
Source: BMC Plant Biol. 2019 Apr 11;19:135. doi: 10.1186/s12870-019-1738-6 (PMC6458719; doi:10.1186/s12870-019-1738-6)

a

	T0/T2 log2Fold Change	FDR	T0/T3 log2Fold Change	FDR	T0/T5 log2Fold Change	FDR	T2/T3 log2Fold Change	FDR	T2/T5 log2Fold Change	FDR	T3/T5 log2Fold Change	FDR
MAF5	-0.72	2.77E-02					0.85	5.69E-04	0.67	3.87E-02		
LNCRNA-MERGE_C-9859	-3.466698	5.65E-05	-2.637844	6.43E-03	-3.433199	1.51E-04	NA	NA	NA	NA	NA	NA

b

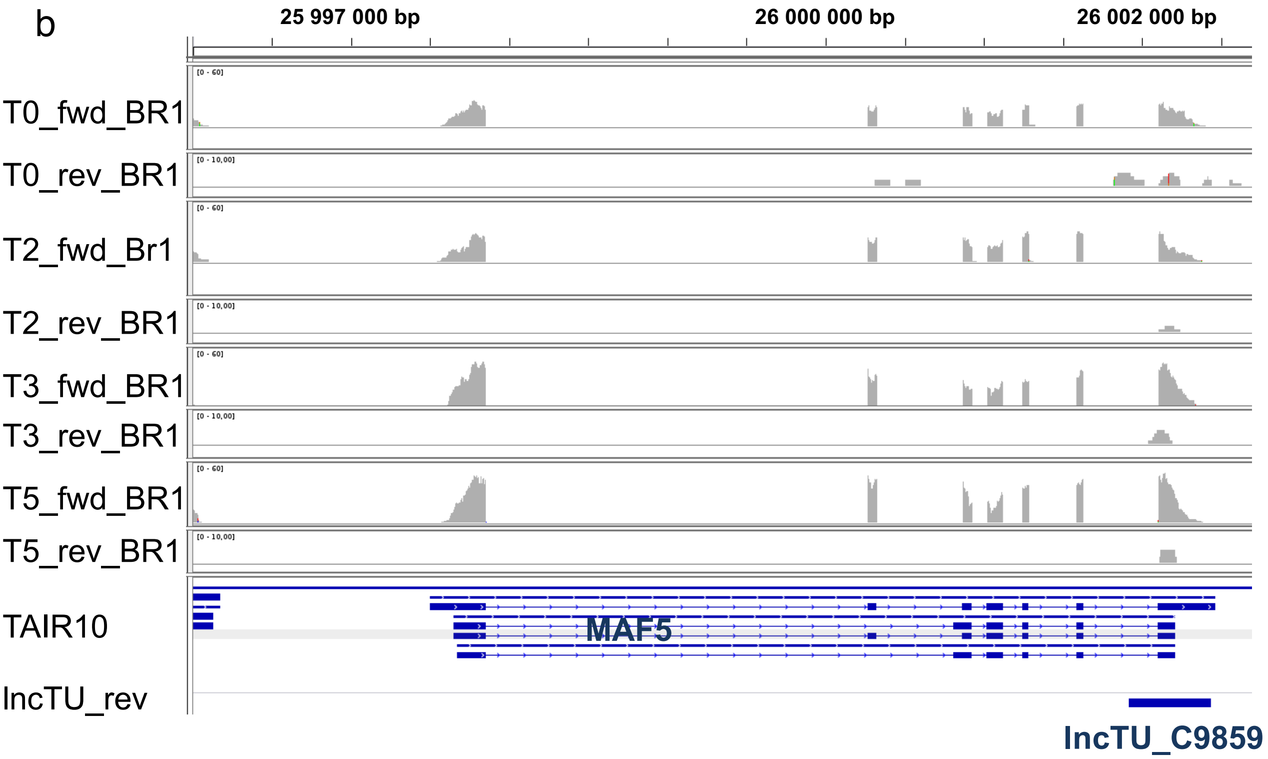

Supplement: Supplementary file 20 — Figure S8. Expression of MAF5 and its antisense lncTU located in the 3′ end of MAF5 region at T0, T2 and T3. (a) Fold changes. (b) Browser snapshot showing the expression profiles. BR1: biological replicate number 1. (PDF 77 kb) [file 12870_2019_1738_MOESM20_ESM.pdf]
